# *catena*-Poly[[bis­(2,4-dichloro­benzoato)bis­(methanol-κ*O*)cobalt(II)]-μ-4,4′-bipyridine-κ^2^ *N*:*N*′]

**DOI:** 10.1107/S1600536811046149

**Published:** 2011-11-09

**Authors:** Min Young Hyun, Pan-Gi Kim, Cheal Kim, Youngmee Kim

**Affiliations:** a Department of Fine Chemistry, Seoul National University of Science and Technology, Seoul 139-743, Republic of Korea; b Department of Forest & Environment Resources, Kyungpook National University, Sangju 742-711, Republic of Korea; c Department of Chemistry and Nano Science, Ewha Womans University, Seoul 120-750, Republic of Korea

## Abstract

In the title compound, [Co(C_7_H_3_Cl_2_O_2_)_2_(C_10_H_8_N_2_)(CH_3_OH)_2_]_*n*_, the Co^II^ ion lies on a twofold rotation axis and is in a slightly distorted octa­hedral CdO_4_N_2_ environment, formed by two O atoms from monodentate dichloro­benzoate ligands, two O atoms from methanol ligands, and two N atoms from *trans*-related 4,4′-bipyridine ligands. The bipyridine ligands also lies on a twofold rotation axis and bridge the Co^II^ ions, forming chains extending along [010]. An intra­chain O—H⋯O hydrogen bond is observed.

## Related literature

For inter­actions of metal ions with amino acids, see: Stoumpos *et al.* (2009 ▶). For related complexes, see: Yu *et al.* (2010 ▶); Hyun *et al.* (2011 ▶); Kang *et al.* (2011 ▶); Kim *et al.* (2011 ▶); Song *et al.* (2009 ▶). 
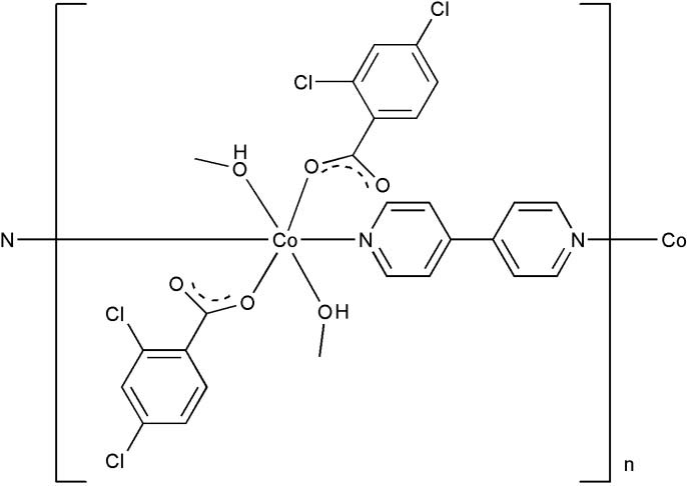


## Experimental

### 

#### Crystal data

[Co(C_7_H_3_Cl_2_O_2_)_2_(C_10_H_8_N_2_)(CH_4_O)_2_]
*M* *_r_* = 659.19
Monoclinic, 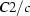

*a* = 20.8405 (16) Å
*b* = 11.4228 (9) Å
*c* = 15.0728 (12) Å
β = 127.479 (1)°
*V* = 2847.5 (4) Å^3^
*Z* = 4
Mo *K*α radiation
μ = 1.02 mm^−1^
*T* = 288 K
0.10 × 0.08 × 0.03 mm

#### Data collection

Bruker SMART CCD diffractometer
Absorption correction: multi-scan (*SADABS*; Bruker, 1997 ▶) *T*_min_ = 0.907, *T*_max_ = 0.970
7806 measured reflections
2801 independent reflections
2178 reflections with *I* > 2σ(*I*)
*R*_int_ = 0.026

#### Refinement

*R*[*F* ^2^ > 2σ(*F* ^2^)] = 0.035
*wR*(*F* ^2^) = 0.092
*S* = 1.06
2801 reflections
184 parameters
H atoms treated by a mixture of independent and constrained refinement
Δρ_max_ = 0.46 e Å^−3^
Δρ_min_ = −0.45 e Å^−3^

### 

Data collection: *SMART* (Bruker, 1997 ▶); cell refinement: *SAINT* (Bruker, 1997 ▶); data reduction: *SAINT*; program(s) used to solve structure: *SHELXS97* (Sheldrick, 2008 ▶); program(s) used to refine structure: *SHELXL97* (Sheldrick, 2008 ▶); molecular graphics: *SHELXTL* (Sheldrick, 2008 ▶); software used to prepare material for publication: *SHELXTL*.

## Supplementary Material

Crystal structure: contains datablock(s) I, global. DOI: 10.1107/S1600536811046149/lh5365sup1.cif

Structure factors: contains datablock(s) I. DOI: 10.1107/S1600536811046149/lh5365Isup2.hkl

Additional supplementary materials:  crystallographic information; 3D view; checkCIF report

## Figures and Tables

**Table 1:** Hydrogen-bond geometry (Å, °)

*D*—H⋯*A*	*D*—H	H⋯*A*	*D*⋯*A*	*D*—H⋯*A*
O3—H3*O*⋯O2^i^	0.70 (3)	1.96 (3)	2.625 (2)	160 (3)

Symmetry code: (i) 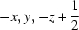.

**Fig. 1.**
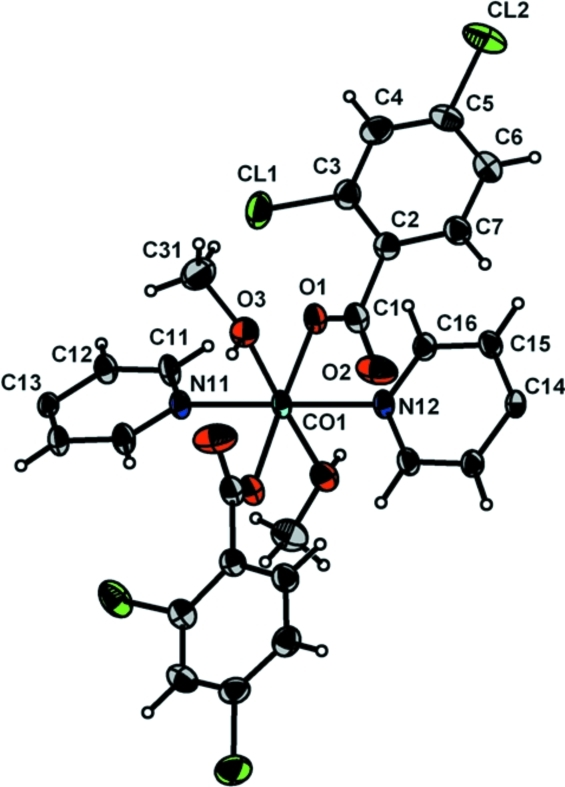
A fragment of the one-dimensional chain structure of the title compound showing displacement ellipsoids at the 30% probability level. Unlabeled atoms are related by the symmetry operator (-x, y, 0.5 - z).

## supplementary crystallographic information

### Comment

The interaction of transition metal ions with biologically active molecules such as amino acids and various acids in the biological systems is of great importance (Stoumpos, *et al.*, 2009). Therefore, we and other groups have intensively examined the interaction of transition metal ions with various acids such as benzoic acid and acetic acid. As a result, there are a variety of structures in the literature of copper(II), cadmium(II), nickel(II), and zinc(II) benzoates with quinoxaline, 6-methylquinoline, 3-methylquinoline, *trans*-1-(2-pyridyl)-2-(4-pyridyl)ethylene, and di-2-pyridyl ketone (Yu, *et al.*, 2010; Hyun, *et al.*, 2011; Kang, *et al.*, 2011; Kim, *et al.*, 2011). Nevertheless, cobalt as a metal ion source has rarely been used (Song, *et al.*, 2009). In this work, we have employed cobalt(II) benzoate as a building block and 4,4'-bipyridine as a ligand. We report herein the crystal structure of the title compound.

In the title compound, the Co^II^ ion lies on a two-fold rotation axis and is in a distorted octahedral CdO_4_N_2_ environment, constructed by two O atoms from dichlorobenzoate ligands, two O atoms from methanol ligands, and two N atoms from the *trans*-related 4,4'-bipyridine, which also lies on a two-fold rotation axis (Fig. 1). The 4,4'-bipyridine ligands bridge the Co^II^ complex units, forming chains extending along the [010] direction.

### Experimental

2,4-Dichlorobenzoic acid (19.1 mg, 0.1 mmol), NH_4_OH (13.9 ml, 0.1 mmol) and Co(NO_3_)2.6H2O (14.6 mg, 0.05 mmol) were dissolved in 4 ml methanol and carefully layered with a 4 ml methylene chloride solution of 4,4'-bipyridine (15.6 mg, 0.1 mmol). Suitable crystals of the title compound were obtained in a month.

### Refinement

H atoms were placed in calculated positions with C—H distances of 0.93-0.96 Å. They were included in the refinement in riding-motion approximation with *U*_iso_(H) = 1.2*U*_eq_(C) or1.5*U*_eq_(C_methyl_). The position of the H atom of the methanol ligand was refined with an isotropic displacement parameter.

### Crystal data

**Table:**

[Co(C_7_H_3_Cl_2_O_2_)_2_(C_10_H_8_N_2_)(CH_4_O)_2_]	*F*(000) = 1340
*M**_r_* = 659.19	*D*_x_ = 1.538 Mg m^−^^3^
Monoclinic, *C*2/*c*	Mo *K*α radiation, λ = 0.71073 Å
Hall symbol: -C 2yc	Cell parameters from 2248 reflections
*a* = 20.8405 (16) Å	θ = 2.3–25.2°
*b* = 11.4228 (9) Å	µ = 1.02 mm^−^^1^
*c* = 15.0728 (12) Å	*T* = 288 K
β = 127.479 (1)°	Plate, orange
*V* = 2847.5 (4) Å^3^	0.10 × 0.08 × 0.03 mm
*Z* = 4	

### Data collection

**Table:**

Bruker SMART CCD diffractometer	2801 independent reflections
Radiation source: fine-focus sealed tube	2178 reflections with *I* > 2σ(*I*)
graphite	*R*_int_ = 0.026
φ and ω scans	θ_max_ = 26.0°, θ_min_ = 2.2°
Absorption correction: multi-scan (*SADABS*; Bruker, 1997)	*h* = −25→18
*T*_min_ = 0.907, *T*_max_ = 0.970	*k* = −11→14
7806 measured reflections	*l* = −18→18

### Refinement

**Table:**

Refinement on *F*^2^	Primary atom site location: structure-invariant direct methods
Least-squares matrix: full	Secondary atom site location: difference Fourier map
*R*[*F*^2^ > 2σ(*F*^2^)] = 0.035	Hydrogen site location: inferred from neighbouring sites
*wR*(*F*^2^) = 0.092	H atoms treated by a mixture of independent and constrained refinement
*S* = 1.06	*w* = 1/[σ^2^(*F*_o_^2^) + (0.0539*P*)^2^ + 0.195*P*] where *P* = (*F*_o_^2^ + 2*F*_c_^2^)/3
2801 reflections	(Δ/σ)_max_ < 0.001
184 parameters	Δρ_max_ = 0.46 e Å^−^^3^
0 restraints	Δρ_min_ = −0.45 e Å^−^^3^

### Special details

**Table:**

Geometry. All e.s.d.'s (except the e.s.d. in the dihedral angle between two l.s. planes) are estimated using the full covariance matrix. The cell e.s.d.'s are taken into account individually in the estimation of e.s.d.'s in distances, angles and torsion angles; correlations between e.s.d.'s in cell parameters are only used when they are defined by crystal symmetry. An approximate (isotropic) treatment of cell e.s.d.'s is used for estimating e.s.d.'s involving l.s. planes.
Refinement. Refinement of *F*^2^ against ALL reflections. The weighted *R*-factor *wR* and goodness of fit *S* are based on *F*^2^, conventional *R*-factors *R* are based on *F*, with *F* set to zero for negative *F*^2^. The threshold expression of *F*^2^ > σ(*F*^2^) is used only for calculating *R*-factors(gt) *etc*. and is not relevant to the choice of reflections for refinement. *R*-factors based on *F*^2^ are statistically about twice as large as those based on *F*, and *R*- factors based on ALL data will be even larger.

### Fractional atomic coordinates and isotropic or equivalent isotropic displacement parameters (Å^2^)

**Table:**

	*x*	*y*	*z*	*U*_iso_*/*U*_eq_	
Co1	0.0000	0.25358 (3)	0.2500	0.03212 (14)	
Cl1	0.14268 (5)	0.07781 (6)	0.63891 (6)	0.0674 (2)	
Cl2	0.27648 (5)	0.39785 (8)	0.96582 (5)	0.0778 (3)	
N11	0.0000	0.06476 (19)	0.2500	0.0357 (6)	
N12	0.0000	0.44441 (19)	0.2500	0.0348 (5)	
O1	0.06270 (9)	0.25755 (11)	0.42246 (12)	0.0390 (4)	
O2	−0.03520 (12)	0.28061 (19)	0.44020 (14)	0.0718 (6)	
O3	0.11240 (11)	0.25907 (14)	0.27604 (15)	0.0460 (4)	
H3O	0.1016 (17)	0.262 (2)	0.222 (2)	0.051 (9)*	
C1	0.03654 (15)	0.27797 (18)	0.47722 (18)	0.0402 (5)	
C2	0.09941 (13)	0.30634 (19)	0.60010 (16)	0.0371 (5)	
C3	0.14937 (15)	0.2225 (2)	0.67935 (19)	0.0435 (5)	
C4	0.20443 (15)	0.2499 (2)	0.79167 (19)	0.0510 (6)	
H4	0.2371	0.1923	0.8439	0.061*	
C5	0.20962 (14)	0.3633 (2)	0.82401 (18)	0.0484 (6)	
C6	0.16244 (15)	0.4504 (2)	0.74806 (19)	0.0485 (6)	
H6	0.1674	0.5274	0.7714	0.058*	
C7	0.10782 (15)	0.42101 (19)	0.63699 (18)	0.0444 (5)	
H7	0.0757	0.4793	0.5852	0.053*	
C11	0.01623 (14)	0.00268 (18)	0.33700 (17)	0.0405 (5)	
H11	0.0275	0.0434	0.3985	0.049*	
C12	0.01723 (14)	−0.11759 (17)	0.34075 (17)	0.0376 (5)	
H12	0.0293	−0.1563	0.4036	0.045*	
C13	0.0000	−0.1806 (2)	0.2500	0.0310 (6)	
C14	0.0000	0.6896 (2)	0.2500	0.0315 (6)	
C15	0.05524 (13)	0.62607 (17)	0.34646 (17)	0.0373 (5)	
H15	0.0929	0.6646	0.4135	0.045*	
C16	0.05389 (14)	0.50533 (17)	0.34212 (17)	0.0371 (5)	
H16	0.0926	0.4643	0.4069	0.045*	
C31	0.17939 (16)	0.1836 (3)	0.3450 (2)	0.0693 (8)	
H31A	0.1901	0.1756	0.4163	0.104*	
H31B	0.2260	0.2159	0.3553	0.104*	
H31C	0.1675	0.1081	0.3101	0.104*	

### Atomic displacement parameters (Å^2^)

**Table:**

	*U*^11^	*U*^22^	*U*^33^	*U*^12^	*U*^13^	*U*^23^
Co1	0.0505 (3)	0.0178 (2)	0.0336 (2)	0.000	0.0284 (2)	0.000
Cl1	0.0934 (6)	0.0445 (4)	0.0610 (4)	0.0235 (3)	0.0453 (4)	0.0071 (3)
Cl2	0.0728 (5)	0.0986 (6)	0.0369 (3)	−0.0283 (4)	0.0205 (3)	−0.0094 (3)
N11	0.0521 (16)	0.0194 (11)	0.0377 (13)	0.000	0.0284 (12)	0.000
N12	0.0500 (15)	0.0205 (11)	0.0388 (13)	0.000	0.0297 (13)	0.000
O1	0.0572 (10)	0.0299 (8)	0.0362 (8)	0.0037 (6)	0.0316 (8)	0.0000 (6)
O2	0.0524 (12)	0.1249 (18)	0.0406 (9)	−0.0081 (11)	0.0295 (9)	−0.0142 (10)
O3	0.0547 (11)	0.0452 (10)	0.0403 (9)	0.0037 (7)	0.0300 (9)	0.0038 (8)
C1	0.0570 (15)	0.0313 (11)	0.0366 (11)	0.0005 (10)	0.0307 (11)	0.0008 (9)
C2	0.0437 (13)	0.0391 (12)	0.0339 (11)	0.0010 (9)	0.0264 (10)	0.0003 (9)
C3	0.0518 (14)	0.0432 (13)	0.0419 (12)	0.0055 (10)	0.0318 (12)	0.0015 (10)
C4	0.0486 (14)	0.0614 (17)	0.0392 (12)	0.0100 (12)	0.0248 (12)	0.0124 (11)
C5	0.0432 (13)	0.0651 (17)	0.0337 (11)	−0.0109 (12)	0.0218 (11)	−0.0053 (11)
C6	0.0557 (15)	0.0466 (14)	0.0466 (13)	−0.0100 (11)	0.0328 (13)	−0.0094 (11)
C7	0.0527 (14)	0.0390 (13)	0.0375 (11)	0.0002 (10)	0.0254 (11)	0.0000 (10)
C11	0.0641 (15)	0.0230 (10)	0.0391 (11)	−0.0017 (10)	0.0339 (11)	−0.0033 (8)
C12	0.0562 (14)	0.0230 (10)	0.0380 (11)	−0.0015 (9)	0.0310 (11)	0.0021 (8)
C13	0.0333 (16)	0.0207 (14)	0.0385 (15)	0.000	0.0216 (14)	0.000
C14	0.0419 (17)	0.0203 (14)	0.0406 (15)	0.000	0.0294 (14)	0.000
C15	0.0468 (13)	0.0254 (10)	0.0363 (11)	−0.0033 (9)	0.0235 (11)	−0.0022 (8)
C16	0.0470 (13)	0.0236 (10)	0.0385 (11)	0.0027 (9)	0.0249 (11)	0.0041 (9)
C31	0.0593 (18)	0.074 (2)	0.0657 (18)	0.0147 (15)	0.0335 (16)	0.0082 (15)

### Geometric parameters (Å, °)

**Table:**

Co1—O1	2.0816 (14)	C4—H4	0.9300
Co1—O1^i^	2.0816 (14)	C5—C6	1.377 (3)
Co1—O3^i^	2.1274 (18)	C6—C7	1.375 (3)
Co1—O3	2.1275 (18)	C6—H6	0.9300
Co1—N11	2.157 (2)	C7—H7	0.9300
Co1—N12	2.180 (2)	C11—C12	1.375 (3)
Cl1—C3	1.738 (2)	C11—H11	0.9300
Cl2—C5	1.744 (2)	C12—C13	1.386 (2)
N11—C11	1.341 (2)	C12—H12	0.9300
N11—C11^i^	1.341 (2)	C13—C12^i^	1.386 (2)
N12—C16^i^	1.333 (2)	C13—C14^ii^	1.484 (4)
N12—C16	1.333 (2)	C14—C15	1.389 (2)
O1—C1	1.257 (3)	C14—C15^i^	1.389 (2)
O2—C1	1.238 (3)	C14—C13^iii^	1.484 (4)
O3—C31	1.417 (3)	C15—C16	1.380 (3)
O3—H3O	0.70 (3)	C15—H15	0.9300
C1—C2	1.516 (3)	C16—H16	0.9300
C2—C3	1.384 (3)	C31—H31A	0.9600
C2—C7	1.392 (3)	C31—H31B	0.9600
C3—C4	1.384 (3)	C31—H31C	0.9600
C4—C5	1.366 (3)		
			
O1—Co1—O1^i^	177.51 (7)	C5—C4—H4	120.7
O1—Co1—O3^i^	90.79 (6)	C3—C4—H4	120.7
O1^i^—Co1—O3^i^	89.13 (6)	C4—C5—C6	121.8 (2)
O1—Co1—O3	89.13 (6)	C4—C5—Cl2	118.86 (19)
O1^i^—Co1—O3	90.79 (6)	C6—C5—Cl2	119.32 (19)
O3^i^—Co1—O3	176.63 (9)	C7—C6—C5	118.7 (2)
O1—Co1—N11	91.25 (4)	C7—C6—H6	120.7
O1^i^—Co1—N11	91.25 (4)	C5—C6—H6	120.7
O3^i^—Co1—N11	91.69 (4)	C6—C7—C2	121.7 (2)
O3—Co1—N11	91.69 (4)	C6—C7—H7	119.2
O1—Co1—N12	88.75 (4)	C2—C7—H7	119.2
O1^i^—Co1—N12	88.75 (4)	N11—C11—C12	123.88 (19)
O3^i^—Co1—N12	88.31 (4)	N11—C11—H11	118.1
O3—Co1—N12	88.31 (4)	C12—C11—H11	118.1
N11—Co1—N12	180.0	C11—C12—C13	119.34 (19)
C11—N11—C11^i^	116.1 (2)	C11—C12—H12	120.3
C11—N11—Co1	121.94 (12)	C13—C12—H12	120.3
C11^i^—N11—Co1	121.94 (12)	C12—C13—C12^i^	117.4 (2)
C16^i^—N12—C16	117.0 (2)	C12—C13—C14^ii^	121.28 (12)
C16^i^—N12—Co1	121.47 (12)	C12^i^—C13—C14^ii^	121.28 (12)
C16—N12—Co1	121.48 (12)	C15—C14—C15^i^	117.0 (2)
C1—O1—Co1	129.08 (15)	C15—C14—C13^iii^	121.48 (12)
C31—O3—Co1	126.90 (17)	C15^i^—C14—C13^iii^	121.48 (12)
C31—O3—H3O	111 (2)	C16—C15—C14	119.49 (19)
Co1—O3—H3O	104 (2)	C16—C15—H15	120.3
O2—C1—O1	126.6 (2)	C14—C15—H15	120.3
O2—C1—C2	117.03 (19)	N12—C16—C15	123.4 (2)
O1—C1—C2	116.4 (2)	N12—C16—H16	118.3
C3—C2—C7	117.4 (2)	C15—C16—H16	118.3
C3—C2—C1	122.9 (2)	O3—C31—H31A	109.5
C7—C2—C1	119.63 (19)	O3—C31—H31B	109.5
C2—C3—C4	121.9 (2)	H31A—C31—H31B	109.5
C2—C3—Cl1	119.86 (18)	O3—C31—H31C	109.5
C4—C3—Cl1	118.28 (18)	H31A—C31—H31C	109.5
C5—C4—C3	118.5 (2)	H31B—C31—H31C	109.5
			
O1—Co1—N11—C11	16.24 (13)	O1—C1—C2—C3	−74.4 (3)
O1^i^—Co1—N11—C11	−163.76 (13)	O2—C1—C2—C7	−71.7 (3)
O3^i^—Co1—N11—C11	−74.59 (13)	O1—C1—C2—C7	106.2 (2)
O3—Co1—N11—C11	105.41 (13)	C7—C2—C3—C4	1.6 (3)
O1—Co1—N11—C11^i^	−163.75 (13)	C1—C2—C3—C4	−177.7 (2)
O1^i^—Co1—N11—C11^i^	16.24 (13)	C7—C2—C3—Cl1	−179.46 (17)
O3^i^—Co1—N11—C11^i^	105.41 (13)	C1—C2—C3—Cl1	1.2 (3)
O3—Co1—N11—C11^i^	−74.58 (13)	C2—C3—C4—C5	−0.7 (4)
O1—Co1—N12—C16^i^	−158.24 (11)	Cl1—C3—C4—C5	−179.63 (19)
O1^i^—Co1—N12—C16^i^	21.76 (11)	C3—C4—C5—C6	−0.7 (4)
O3^i^—Co1—N12—C16^i^	−67.41 (12)	C3—C4—C5—Cl2	177.98 (18)
O3—Co1—N12—C16^i^	112.59 (12)	C4—C5—C6—C7	1.1 (4)
O1—Co1—N12—C16	21.76 (11)	Cl2—C5—C6—C7	−177.58 (18)
O1^i^—Co1—N12—C16	−158.24 (11)	C5—C6—C7—C2	−0.1 (4)
O3^i^—Co1—N12—C16	112.59 (12)	C3—C2—C7—C6	−1.2 (3)
O3—Co1—N12—C16	−67.41 (12)	C1—C2—C7—C6	178.2 (2)
O3^i^—Co1—O1—C1	−11.09 (17)	C11^i^—N11—C11—C12	0.28 (17)
O3—Co1—O1—C1	165.54 (17)	Co1—N11—C11—C12	−179.71 (17)
N11—Co1—O1—C1	−102.79 (16)	N11—C11—C12—C13	−0.6 (3)
N12—Co1—O1—C1	77.21 (16)	C11—C12—C13—C12^i^	0.27 (16)
O1—Co1—O3—C31	52.8 (2)	C11—C12—C13—C14^ii^	−179.74 (16)
O1^i^—Co1—O3—C31	−129.7 (2)	C15^i^—C14—C15—C16	1.09 (14)
N11—Co1—O3—C31	−38.4 (2)	C13^iii^—C14—C15—C16	−178.90 (14)
N12—Co1—O3—C31	141.6 (2)	C16^i^—N12—C16—C15	1.19 (15)
Co1—O1—C1—O2	13.4 (3)	Co1—N12—C16—C15	−178.81 (15)
Co1—O1—C1—C2	−164.25 (13)	C14—C15—C16—N12	−2.4 (3)
O2—C1—C2—C3	107.7 (3)		

Symmetry codes: (i) −*x*, *y*, −*z*+1/2; (ii) *x*, *y*−1, *z*; (iii) *x*, *y*+1, *z*.

### Hydrogen-bond geometry (Å, °)

**Table:**

*D*—H···*A*	*D*—H	H···*A*	*D*···*A*	*D*—H···*A*
O3—H3O···O2^i^	0.70 (3)	1.96 (3)	2.625 (2)	160 (3)

Symmetry codes: (i) −*x*, *y*, −*z*+1/2.
